# 

**Published:** 1897-06

**Authors:** 


### NOTICES.

### New Jersey State Dental Society.

The twenty-seventh annual meeting of the New Jersey State Dental Society will be held in Atlantic City, on July 21st to 23d, inclusive.

The Committee on Exhibits have secured four connecting rooms on first floor of the Grand Atlantic Hotel. Everything in connection with dental meeting can be accommodated under the one roof.

The hotel is located in the centre of the block, with all sides available for light and ventilation, which insures a cool and comfortable meeting place at all times.

The hotel is new, with all improvements—electric light power, telephone and telegraph. Also a bureau of information with attendant, and a private watchman to look after the interests of the exhibitors.

A plan of rooms is hereby submitted, showing the allotted table spaces. The Committee have received a number of calls for space, even before the rooms have been secured, and they would suggest to those desiring location, to communicate with the Chairman as soon as possible, marking upon the plan four or five spaces most desirable to them, that one of the locations selected may, without a doubt, be secured.

Those engaging space prior to the programme going to print will be mentioned therein.

Only one restriction is made by the Committee: *All goods must be covered during meeting hours.*

When the desired location is secured, price of the same must be deposited, or space will not be reserved.

Exhibition tables are four feet wide. The length will be according to space upon the floor. Price $1.50 per running foot for any length less than six feet. Six feet and over, $1.25 per running foot. Nothing less than four feet will be sold.

J. L. Crater, Chairman, Orange.
William P. Richards, Orange.
C. W. F. Holbrook, Newark.
F. E. Riley, Newark.
H. S. Sutphen, Newark.
C. S. Hardy, Summit.
*Exhibit Committee.*

### American Dental Association.

The thirty-seventh annual session of the American Dental Association will be held at Old Point Comfort, Va., commencing at 10 A. M., on Tuesday, August 3d, 1897.

Geo. H. Cushing, *Recording Secretary.*

### The Indiana State Dental Meeting.

The Indiana State Dental Association will hold its thirty-ninth annual meeting in Fort Wayne, commencing at 10 o'clock A. M., June 29th and continuing through three days. Members of the profession are cordially invited to be present and participate in the proceedings.

Monday, June 28th, at 2 P. M., the State Board of Dental Examiners will meet in the New Aveline Hotel to examine all applicants coming before it.

M. A. Mason, D. D. S., *Secretary.*

FORT WAYNE, IND., May 18, 1897.

*Dear Doctor:*

The next meeting of the Indiana State Dental Association will be held in this city June 29th, 30th and July 1st. It is expected that this meeting will be of unusual interest and all in attendance will have a time of great profit and enjoyment. Our railroad facilities will enable a large number of our dentists to reach here without much trouble.

If there is anyone who ought to appreciate the value of a dental meeting with its social features, its papers and discussions, its clinics, etc., it is the graduate; and who is better prepared to drink in and assimilate all the valued things offered at our dental societies, than the graduate of that par-excellent institution, the University of Michigan.

In our State is a large number of graduates of the Dental Department of the University of Michigan, who desire that arrangements be made for a re-union of the alumni, at the time of our State meeting. Consequently this call. Come and meet your old college friends and talk over old times, and if possible, join our Association. At any rate, join us in a re-union. Please reply to this letter. Yours respectfully,

S. B. HARTMAN, '77,
E. FRANK SITER, '79,
M. A. MASON, '90, *Secretary.*

### EDITORIAL.

### To Old Point Comfort.

We have had a number of inquiries lately, as to the best route by which to go from Cincinnati and all the country round about, to the dental meetings to be held about August 1st.

To give a broader answer to this question than can be given to a few individual inquirers, we will here say, that for those wishing to go most directly and most comfortly, with the shortest miles of travel, will attain their full desire by taking the Chesapeake & Ohio road.

Of the comfort and pleasure of travel by this route, nothing need be said here to those who have had experience with its comfort, perfect management and delightful scenery through which it goes; and to those who have not traveled this route, we are fully warranted in saying that it has no superior in all these particulars. It runs directly through from Cincinnati to Old Point Comfort without break or change. unless the passenger wishes to do so. It is the only line making such connection.

If we were going to Boston, we would not likely take this route; if to New York or Philadelphia, we might or might not; if however to Old Point Comfort, Washington City, or Baltimore, the Chesapeake & Ohio is the road, *par excellence.*

We speak from many experiences. In traveling over this road one avoids the fatigue, weariness and monotony that is sometimes the *bete noir* of travel.

### Meetings of The American Dental Association and The Southern Dental Association.

The annual meetings of these bodies will be held at Old Point Comfort, beginning on Tuesday, Aug. 3rd, at 10 o'clock A. M.

These will be by far the most important meetings that these representative bodies have ever held. The question of uniting them into one national body has been considered for a number of years, and from time to time steps have been taken looking to that end, though nothing of a radical character was done, that seemed to be bringing them more nearly into harmony. The condition of affairs that existed for many years in by-gone days seemed hardly propitious for such a union. These conditions have, however, been greatly modified, difficulties removed, harmony established where discord at one time prevailed, and to such a degree has this been accomplished that it would seem there is scarcely any longer an occasion for separate organization. The work of each in their separate spheres has been quite fully accomplished. This, I think, is fully acknowledged by all who are well acquainted with the history of these bodies and with all such there will probably be no objection.

Undoubtedly, the interest of the dental profession will be greatly subserved by such a union. A national body of this kind can far more efficiently utilize its directing and fostering hand in the organization and support of local societies all over the country than was possible under the conditions heretofore existing.

Neither one of these bodies has had the full and complete support of the entire profession, and so could not possibly carry forward any work in which they might wish to engage as if the whole force were combined in one effort. It will be possible, and we think practicable for the united body to exercise a far greater and more beneficial influence upon the educational work of the profession than has been possible in the past. In regard to dental legislation the same thing will hold true.

The one great want that has existed hitherto is lack of uniformity in the laws of our different States regulating the practice of dentistry, and one body, embracing the whole profession, could undoubtedly act more efficiently than two separate bodies have done.

The importance of these questions is so great that every member of these bodies ought to exercise his best effort for unification. We are not fully advised as to what the committees having this matter in charge have done. We have some intimations, however, that they have been industriously at work, and there is great hope entertained that the result of that work may be the immediate union of these two bodies.

We can conceive of no obstacle that ought for a moment longer to keep these two bodies apart, and every consideration is decidedly in favor of their union.

### Meeting of the National Association Dental Faculty.

The annual meeting of this body will be held at the Hygeia Hotel, Old Point Comfort, beginning on Thursday, July 29th, 1897. The Executive Committee will meet at 10 A. M. for the preparation of business for the general session which will open at 2:30 P. M. the same day and at the same place.

This will probably be one of the most interesting and important meetings this body ever held. It will hold one session each day alternately with the Technich Association.

The precise hours for each of these will be determined at the time.

It is requested that any parties having questions or matters to bring before the body, and that should have some consideration before presentment to the body should send the same to Dr. B. Holly Smith, 1007 Madison Ave., Baltimore, Md., at least one month before the meeting.

It is to be hoped that at this meeting or in the near future a union of the N. A. D. F. and the Technich Association may be affected.

As the work of the two bodies is along the same line, namely the educational, and could certainly be more effective under a general than under a divided guidance, it is to be hoped that all the membership of this Association will be fully represented at the opening meeting, that the work may proceed with promptness and efficiency from the beginning.

